# P2TF: a comprehensive resource for analysis of prokaryotic transcription factors

**DOI:** 10.1186/1471-2164-13-628

**Published:** 2012-11-15

**Authors:** Philippe Ortet, Gilles De Luca, David E Whitworth, Mohamed Barakat

**Affiliations:** 1 CEA, DSV, IBEB, SBVME, LEMiRE, Saint-Paul-lez-Durance, F-13108, France; 2 CNRS, UMR 7265, Saint-Paul-lez-Durance, F-13108, France; 3 Aix-Marseille Université, Saint-Paul-lez-Durance, F-13108, France; 4 Institute of Biological, Environmental and Rural Sciences, Aberystwyth University, Ceredigion, UK

## Abstract

**Background:**

Transcription factors (TFs) are DNA-binding proteins that regulate gene expression by activating or repressing transcription. Some have housekeeping roles, while others regulate the expression of specific genes in response to environmental change. The majority of TFs are multi-domain proteins, and they can be divided into families according to their domain organisation. There is a need for user-friendly, rigorous and consistent databases to allow researchers to overcome the inherent variability in annotation between genome sequences.

**Description:**

P2TF (Predicted Prokaryotic Transcription Factors) is an integrated and comprehensive database relating to transcription factor proteins. The current version of the database contains 372,877 TFs from 1,987 completely sequenced prokaryotic genomes and 43 metagenomes. The database provides annotation, classification and visualisation of TF genes and their genetic context, providing researchers with a one-stop shop in which to investigate TFs. The P2TF database analyses TFs in both predicted proteomes and reconstituted ORFeomes, recovering approximately 3% more TF proteins than just screening predicted proteomes. Users are able to search the database with sequence or domain architecture queries, and resulting hits can be aligned to investigate evolutionary relationships and conservation of residues. To increase utility, all searches can be filtered by taxonomy, TF genes can be added to the P2TF cart, and gene lists can be exported for external analysis in a variety of formats.

**Conclusions:**

P2TF is an open resource for biologists, allowing exploration of all TFs within prokaryotic genomes and metagenomes. The database enables a variety of analyses, and results are presented for user exploration as an interactive web interface, which provides different ways to access and download the data. The database is freely available at http://www.p2tf.org/.

## Background

Transcription factors (TFs) are DNA-binding proteins involved in the regulation of gene expression. They are found in all living organisms and activate or repress transcription by binding to specific DNA sequences. TFs are characterized by their DNA-binding domains (DBDs), of which the helix-turn-helix (HTH) domain is the most prevalent in prokaryotic genomes [1].

Many TFs are constitutively active and used to regulate gene expression by changing their levels inside the cell. For example, CarA of *Myxococcus xanthus* is a repressor of photoprotective carotenoid biosynthesis, and illumination results in the production of an anti-repressor which prevents CarA binding to its operator site [2]. Other TFs (known as one-component systems, or OCSs) are switched on/off by the activity of a sensory domain within the protein, for instance LexA of *Escherichia coli* is an inhibitor of the SOS response genes, until binding to activated RecA leads LexA to autoproteolyse [3]. More typically, the activity of OCS sensory domains is regulated by small molecule binding, for instance the PurR regulator acts as repressor of purine biosynthesis upon binding a purine co-repressor [4].

Another common form of TF is the response regulator (RR), which has an N-terminal phospho-acceptor domain. TF activity of the RR is regulated by the phosphorylation state of the RR, which is governed by an environmentally-sensitive histidine kinase. For example, GacA is a pleiotropic regulator in Pseudomonadales, whose activity is modulated through its phosphorylation by the histidine kinase GacS [5]. A histidine kinase-RR pair is known as a two-component system, and these signalling pathways are abundant in prokaryotes. The final major subset of TFs is sigma factors (SFs), which are eubacterial transcription initiation factors. SFs are a labile component of the RNA polymerase holoenzyme which direct the polymerase to specific subsets of promoters. SFs are often regulated by anti-sigma factors: environmentally sensitive inhibitors of SF activity. For instance in *M. xanthus* the anti-SF CarR holds the SF CarQ inactive until cells are illuminated, and then CarQ is released to mediate expression of carotenoid expression [6].

TFs can be categorized into OCSs, RRs, and SFs (and sub-families thereof) through analyses of domain architecture. We defined as Transcriptional Regulators (TRs), a fourth category of TFs, which are not OCSs, RRs or SFs. In addition to domain composition, gene organisation in the vicinity of a TF gene can be important for understanding the function of the TF. Generally TFs tend to be located in the genome adjacent to genes whose expression they regulate (which often includes their own gene). Additionally, many TFs are regulated by adjacently encoded gene products such as histidine kinases (which regulate RRs), and anti-SFs (which regulate SFs). Thus a full computational analysis of TFs needs to provide information on both their domain architecture and also their gene neighbourhood.

Completely sequenced prokaryotic genomes continue to become available at an ever-increasing rate in the primary databases, and as genome annotation standards still differ widely, there is also an escalating need for secondary databases which undertake rigorous and consistent analysis across all available genomes.

Of prokaryotic TF-related databases, ArchaeaTF contains data from a specific kingdom [7], a genome-wide survey of TFs in prokaryotes analyses a subset of available genomes [8] and DBD identifies TFs among a set of predicted (and experimentally defined) proteins [9]. However, to our knowledge, there is no database that provides detailed information about mis-predicted or metagenomic TFs for all completely sequenced genomes and metagenomes.

We have constructed P2TF (Predicted Prokaryotic Transcription Factors) a user-friendly database of predicted TFs for all available completely sequenced prokaryotic genomes and metagenomes. In order to reliably detect TFs, we developed a method analysing TFs in both predicted proteomes and reconstituted ORFeomes, recovering 3% more TFs. P2TF also presents a thorough analysis of the properties of each predicted TF and implements a hierarchical classification scheme. The set of TFs in P2TF can be filtered in a variety of ways (eg. by taxonomy, domain architecture, family membership, gene organisation etc.), and user-defined subsets of TFs can be outputted in a range of formats, to maximise its usefulness to the community. The result of the P2TF analysis process applied to microbial genomes and metagenomes is accessible via a web interface designed primarily for experimental biologists, at http://www.p2tf.org.

## Construction and content

**Figure 1:**
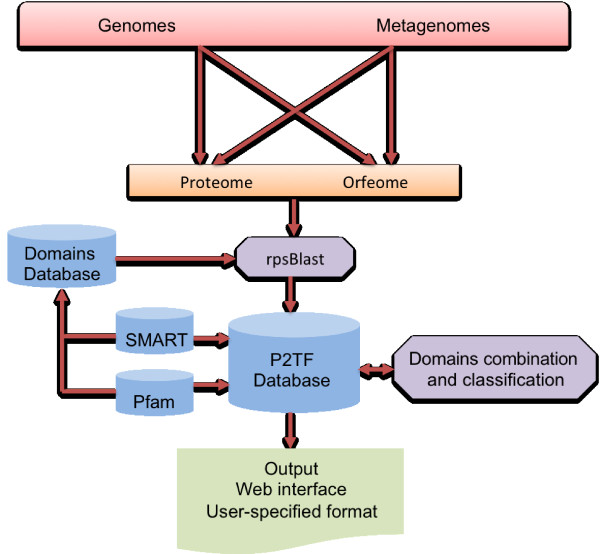
**Flowchart of the P2TF process.** Proteomes are screened for the presence of conserved domains using SMART and Pfam domain profiles. Proteins with hits to appropriate domain profiles are then assigned to specific categories and sub-categories of TFs, and annotated with domain architecture and classification results. The results of the TF analysis can then be viewed as an interactive webpage or exported in a user-defined format.

An overview of the P2TF process is provided in Figure 1. Genome data were imported from the NCBI (National Center for Biotechnology Information) and metagenomic data were downloaded from the IMG/M (Integrated Microbial Genomes with Microbiome Samples). Based on analysis of 1,987 completely sequenced genomes and 43 metagenomes, a pipeline was designed to take protein and whole replicon genomic files as input. Constitution of ORFeomes in the P2TF database is performed as described previously [10]. In brief, each DNA sequence is scanned to predict the pool of valid ORFs, which in turn are translated to constitute an ORFeome. This approach increases the number of TFs identified by approximately 3%. TFs identified in this way are described in P2TF as ‘mispredicted’ proteins (ie. not present in the original genome proteome file) and exclude gene products originally annotated as pseudogenes. Overall, for each genome, the pseudogenes are distinguished in black on the genomic context image, to facilitate their visualisation.

P2TF predicts TF candidates by performing a domain analysis of each protein sequence using RPS-BLAST and considering an E-value cut-off of 0.01 and minimum alignment coverage of 50% for each domain length. We manually selected a pool of domains from the Pfam [11] and SMART libraries [12], based on analysis of the literature on sequence-specific DBDs and their associated domains [8,9,13] (See documentation page of the database and Additional file 1). The presence in a protein of a domain defined as a DBD leads to inclusion of the protein in P2TF. In rare cases experimentally-validated TFs will not be found in P2TF because they possess relatively novel DNA-binding sequences which have not yet been ascribed a domain profile by Pfam or SMART. When such novel DNA-binding domain profiles are published they will be added to P2TF allowing recovery of further TFs.

**Figure 2:**
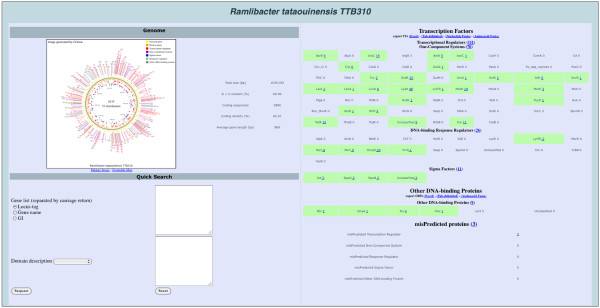
**Genome page of *Ramlibacter tataouinens*is.** In addition to displaying categorization, links are provided to a detailed gene list. Clickable links are underlined. P2TF provides also a depiction of the distribution of TF and ODP genes around the replicon generated using CGView [18].

The P2TF analysis then divides DBDs into TFs and ‘Other DNA-binding Proteins’ (ODPs), a category, which includes non-regulatory DNA-binding proteins such as tranposases, integrases and histone-like proteins. TFs are then further divided into sub-categories (TRs, OCSs, RRs and SFs) according to their domain architecture (Additional file 2). TFs, which contain a CheY-like receiver (phosphoacceptor) domain, are annotated as RRs. These proteins form, with partner sensor histidine kinases, two-component systems [14,15], which are specifically analysed elsewhere [10,16]. SFs were divided into 3 sub-groupings (families); RpoN, RpoD (including housekeeping SFs) and ECF (extra-cytoplasmic function) SFs. RpoN proteins contained Sigma54 DNA-binding and core-binding domains (pfam4552 and pfam4963), ECF SFs were defined as proteins containing Sigma70 regions 2 and 4 (pfam4542 and pfam4545), while RpoD proteins also contained region 3 (pfam4539). OCSs were defined as proteins carrying both input and output domains but lacking phosphotransfer domains characteristic of two-component systems (as defined by [17]). TRs, OCSs and RRs are also sub-divided into 71 distinct sub-groupings (families) depending upon which domains were present in the proteins. For instance MerR family OCSs contain MerR DBD and B12-binding domains. For each genome a summary page is provided showing the results of the classification process (Figure 2).

Users are encouraged to suggest modifications to the database such as the creation of new categories or families, and/or to validate/curate predicted proteins. To ensure integrity of the database, we ask interested experts to download formatted data from P2TF and then after manual curation, the same downloaded files can be used as an exchange format for updating the database. Four genomes have already been manually curated by the authors (*Ramlibacter tataouinensis*, *Pseudomonas brassicacearum*, *Deinococcus deserti* and *Myxococcus xanthus*).

The P2TF database can be queried via two modes: keyword searches, and BLAST searches. The first search mode allows users to request genes on the basis of their locus-tag, gene name, GI (GenBank Identifier) or domain possession. To restrict their search, users can browse predictions by querying a genome of interest or a group of genomes belonging to the same taxon. A taxonomic search can be achieved by using the species name, the taxon-id or the lineage name.

A BLAST search mode was also implemented to provide a similarity search against all or individual species within P2TF. Users can use default BLAST parameters or simply modify these by entering new options. In addition users can use P2TF proteins as queries for BLAST searches for similar proteins in Uniprot or Genbank through a link on each P2TF protein page.

The search modules build search output as a tabular view that is linked to a full description and genomic context for each selected gene. A selection system has been implemented to add all or partial data to a shopping cart or perform a multiple sequence alignment using the MUSCLE program [19]. The resulting multiple sequence alignments can then be viewed using the Jalview applet [20].

P2TF also provides information regarding TF gene organisation both as a CGView [18] zoomable genome map on each genome page, and as a zoomable genome browser linked from each gene page. The prediction of putative regulation regions is provided by considering the flanking genes of the current TF within 500 bp of the TF of interest. The categorization system uses the Cluster of Orthologous Groups (COG) classifications [21] to define non-TF genes.

## Utility and discussion

### Overview

**Figure 3:**
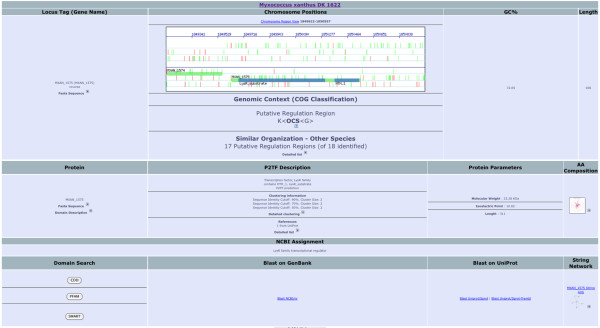
**A LysR family TF of *M. xanthus *as displayed in P2TF.** A comprehensive description of the protein, including published literature through STRING and UniProt, a cartographic gene context (Chromosome Region View), with several options such as zooming in or out, moving along the chromosome, displaying genes in upstream or downstream regions and drawing genes and several external links to perform blast searches against several databases.

Extraction of relevant biological information from the huge amount of available genomic and metagenomic sequence data is still a formidable challenge. Indeed, biologists with little or no computing background have an increasing need for fast and intuitively usable tools, which is why P2TF has been developed as an interactive system for editing and viewing TF information, to help increase understanding of transcription regulation processes. P2TF differs in many ways from other public TF databases. It includes all available completely sequenced prokaryotic genomes, and also features metagenomic data, ORFeomes are scanned as well as proteomes, detailed information is provided for each TF, multiple query-routes are available including sequence-based queries, results can be formatted by taxonomy and aligned, and data can be outputted for download in a variety of user-specified formats. The current version of P2TF contains 372,877 DBD proteins, from 1,987 genomes and 43 metagenomes (6% from metagenomes), and includes 205,290 TRs, 81,870 OCSs, 38,032 RRs, 24,240 SFs and 23,445 ODPs. A comprehensive description of each protein within P2TF is provided (Figure 3), including published literature through UniProt and STRING [22,23], and a link for each reference to the PubMed database. The collected data can be freely downloaded from our server in a variety of formats, including Excel, nucleotide FASTA, amino acid FASTA and tab-delimited.

The ORFeome search is an important feature of the P2TF process, which on average allows the recovery of nearly 3% more TFs per replicon. However some replicons feature extremely large numbers of mispredicted TF genes. For instance, 147 TFs and ODPs were identified in the proteome of *Sodalis glossinidius* str. ‘morsitans’, but a further 100 (68% of the original complement) were found by searching its ORFeome.

To assess the performance of P2TF, we compared P2TF data (not including ORFeome results) with datasets derived for four genomes (*Bacillus subtilis* 168, *Lactobacillus acidophilus* NCFM, *Stenotrophomonas maltophilia* R551-3 and *Corynebacterium efficiens* YS-314), from three studies [9,24,25]. We considered sensitivity (true positives/(true positives + false negatives)) and specificity (true negatives/(true negatives + false positives)). In all four comparisons specificity was greater than 98%, while the mean sensitivity was 94% (Table 1).

**Table 1:** Performance statistics for P2TF

**Species**	**Reference TFs**	**P2TF TFs**	**Sensitivity (%)**	**Specificity (%)**	**Reference**
*Bacillus subtilis* 168	*237*	*280*	*91.6*	*98.4*	Moreno-Campuzano *et al., 2006*
*Lactobacillus acidophilus* NCFM	*93*	*107*	*93.5*	*98.9*	Wilson *et al*., 2008
*Stenotrophomonas maltophilia* R551-3	*262*	*292*	*93.9*	*98.8*	Wilson *et al*., 2008
*Corynebacterium efficiens* YS-314	*103*	*150*	*97.1*	*98.3*	Brune *et al.,* 2005

Data from P2TF were compared with the results of previously published studies for four bacterial genomes.

The seven bacteria with the largest number of TFs are all Actinomycetes, one of the best-represented phyla among completely sequenced bacterial genomes. In particular, the genomes of five Actinomycetes (*Streptomyces bingchenggensis* BCW-1, *Amycolatopsis mediterranei* S699, *Amycolatopsis mediterranei* U32, *Streptomyces violaceusniger* Tu 4113 and *Streptosporangium roseum* DSM 43021) each harbour more than 1000 DBD-proteins, and the 806 DBD-proteins in the proteome of another Actinomycete (*Kribbella flavida* DSM 17836) account for 11.6% of its total protein-coding genes. The relative and absolute numbers of OCSs, RRs, SFs and other TRs varies dramatically between genomes. For the 1,227 organisms whose genomes contain 100 or more DBD proteins, *Burkholderia* sp. possess the greatest numbers of OCSs at the expense of SFs, while Actinobacteria such as *Catenulispora* sp. possess large numbers of SFs, and Firmicutes (such as *Paenibacillus* sp.) possess relatively large numbers of RRs. The Archaebacteria (such as *Solfolobus* sp.) tend to rely heavily on TRs for gene regulation, with most Archaebacterial genomes completely lacking SFs and RRs.

### Querying P2TF

A search engine was developed that allows users to request genes on the basis of keywords, including locus-tag, gene name, GI or domain possession. Users can also restrict their search to a genome of interest or a group of genomes belonging to the same taxon. In addition, the database can be queried through a BLAST search, enabling users to identify homologs of a query sequences and to perform multiple sequence alignments using the MUSCLE program. The resulting multiple sequence alignments can then be viewed, edited and analysed through the incorporation of the Jalview applet. For instance alignments can be edited, coloured, sorted and outputted for publication, while phylogenetic trees can also be produced from the alignment, providing insights into evolution and the conservation of amino acid residues.

**Figure 4:**
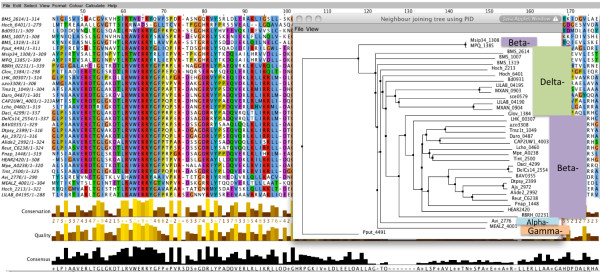
**Phylogenetic relationships between B12-binding domains of Proteobacterial TFs.** A screenshot of a neighbour-joining tree (foreground), with the Jalview sequence alignment in the rear window. Sequences are highlighted as members of the Alpha-, Beta-, or Delta- subgroups of the Proteobacteria.

As an example, CarA (MXAN_0903) of *M. xanthus* possesses a MerR DBD, and a C-terminal B12-binding domain. A P2TF query of Proteobacterial TFs containing B12-binding domains revealed 37 proteins, 35 of which possessed an additional MerR DBD as for CarA (12 Delta subgroup, 20 Beta subgroup, 2 Gamma and 1 Alpha subgroup - *Agrobacterium vitis*). A phylogenetic tree of the B12-binding domains of the proteins shows that the Alphaproteobacterial proteins are most closely related to the Deltaproteobacterial sequences (Figure 4), suggesting there may have been lateral transfer of these genes between ancestral Delta- and Alpha- Proteobacteria. In addition, the CarA gene of *M. xanthus* appears to have undergone a lineage-specific duplication, creating a homologue adjacent in the genome (MXAN_0904).

### Domain architecture analysis

The complement of domains within a protein provides information regarding its biological function, mode of action, and evolutionary heritage. We developed a categorization system, which allocates membership of TFs to families as a consequence of their domain architecture. Thus CarA is a member of the MerR family as it contains a MerR DBD.

Proteins with multiple DNA-binding domains are well characterised in eukaryotes, for instance HMG proteins and Pax/homeodomain proteins [26,27]. In such proteins the multiple DNA-binding domains can result in DNA cross-linking and chromatin compaction, and can enable both specific and modular regulation of gene expression by single proteins. Park *et al.*[28], found that in fungal genomes many TFs carry more than one type of DNA-binding domain, suggesting they interact with multiple regulatory sequences. This also seems to be the case for prokaryotes, as P2TF identifies TFs, which possess multiple DNA-binding domains, in many cases of different types (where TFs contain more than one type of DNA-binding domain, they are assigned to a family in P2TF according to the domain which gives the lowest E-value). This was expected for SFs, which generally have two different DBDs, but not for others TFs. For instance MXAN_0631 and ABC0986 have domain architectures of HTH_8, HTH_AraC and HTH_IclR, Mga respectively. Two proteins from *Desulfotomaculum kuznetsovii* DSM 6115, Desku_0047 and Desku_2016, possess 3 DNA-binding domains, all of the same type (HTH_Xre). We were unable to find examples in the literature of experimentally-characterised prokaryotic TFs which contain multiple DNA-binding domains. The observation of large numbers of such proteins encoded in bacterial genomes suggests that they represent a commonplace but unappreciated group of regulators in these organisms.

There is a strong taxonomic bias affecting the families of TFs that are found in particular genomes. For taxonomic classes with 100 or more DBD proteins across their sequenced genomes, some TF families were found to be unusually common in particular classes. For instance 26% of the TFs in the Epsilonproteobacteria belong to the OmpR family, 18% of Methanomicrobia TFs are ArsR family members, 25% of Betaproteobacterial TFs are LysR, and 19% of TFs in Actinobacteridae genomes belong to the TetR family. Even at lower taxonomic levels there are considerable differences between genomes in terms of their TF family composition. Within the class Actinobacteria, 19% of genus *Bifidobacterium* TFs belong to the LacI family, although for all other Actinobacterial genera LacI TFs represent less than 4% of TFs. Within the Gammaproteobacterial class, LuxR family TFs account for 17% of *Photorhabdus* and *Xenorhabdus* TFs, but at most 6% of the TFs in other Gammaproteobacterial genera.

The OCS LexA is involved in DNA repair as part of the SOS response in *E. coli*[29]. Most of the LexA family TFs contain a LexA_DNA_bin domain and another domain - Peptidase_S24 (pfam00717, which was not originally specified in P2TF). Searches for TFs in P2TF containing the Peptidase_S24 domain gave 1978 TFs. 1078 were associated with a LexA_DNA_bin domain (and therefore classified as members of the LexA family). The other TFs were associated with HTH_XRE or HTH__3 (Xre family) domains, and many of these are known to be true LexA repressors, for instance in the radiation resistant bacterium *Deinococcus radiodurans*[30]. Therefore it seems that the identification of LexA repressors is most probably linked to the association of a Peptidase_S24 domain with any DBD, not just to the LexA_DNA_bin domain. HTH_XRE and HTH_3 domains are very similar (98%), so proteins with either domain are now grouped together in P2TF within the Xre family. Similarly any proteins with a DBD and a peptidase_S24 domain are now classified as LexA proteins.

## Conclusions

Experimental biologists need diverse types of information to guide their experiments and to interrogate large –omics datasets. For instance lists of TFs within a genome can be vital in identifying candidate genes for disruption, for shortlisting potentially redundant orthologues, and thus for infering the genetic mechanism underlying observed phenotypic data. In addition the ability to routinely generate –omics datasets means that experimental biologists need tools for the rational exploration of –omics datasets – for instance by filtering data against rigorously-defined subsets of TFs. In addition, understanding the evolutionary events, which have led to contemporary sets of proteins can provide important insights into the functioning of contemporary regulatory networks, especially by identifying strain- and species-specific changes in TF complements. The P2TF resource provides data and analysis tools, which can assist all such applications and many more besides.

P2TF is an open resource for biologists and data are presented for user exploration as an interactive web interface. The database can be queried in a variety of ways – keyword and sequence based searches are enabled in addition to browsing, and the resulting data can be outputted in user-specified formats. Results from BLAST and domain architecture searches can be aligned using MUSCLE and the alignments edited, viewed and analysed with Jalview. P2TF uses a domain architecture-based classification scheme to categorize TFs into families, and a wide range of information is provided for each P2TF entry including PubMed links. A P2TF cart is also provided to assist analysis by users. P2TF thus provides experimental biologists and bioinformaticians with a high-quality dataset for the investigation of prokaryotic TFs.

The earlier description of how the P2TF LexA classification criteria developed illustrates how users can have input into the functionalities of P2TF. Innovations to the current P2TF scheme such as the inclusion of new domain profiles, and changes to the classification scheme can be readily implemented at the request of users. The developers hope that the user community will help develop future iterations of P2TF for everyone’s benefit.

## Availability and requirements

P2TF is publicly available at http://www.p2tf.org, and runs on all web browsers tested, including Mozilla Firefox, Internet Explorer, Google Chrome and Apple Safari.

## Competing interests

The authors declare that they have no competing interests.

## Authors’ contributions

MB and PO developed and designed the database. GD and DW participated in the improvement of the database functionalities. GD and DW validated model strains data. MB and DW drafted the manuscript. GD and PO revised the manuscript. All authors have read and approved the final submitted version of this manuscript.

## Supplementary Material

Additional file 1 Classification of TF categories: Schematic representation of the conserved domain architectures. Click here for file

Additional file 2 Classification of TF families: List of domain architectures. Click here for file
