# Supplementary material for: P2TF: a comprehensive resource for analysis of prokaryotic transcription factors
Source: BMC Genomics. 2012 Nov 15;13:628. doi: 10.1186/1471-2164-13-628 (PMC3532121; doi:10.1186/1471-2164-13-628)
Supplement: Additional file 2 — Classification of TF families: List of domain architectures. [file 1471-2164-13-628-S2.pdf]

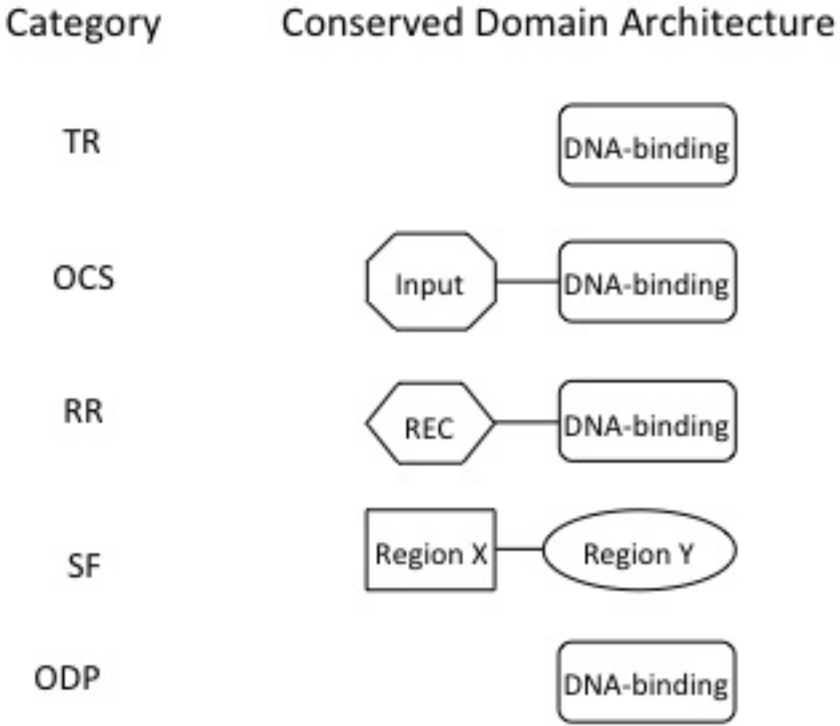
Category
Conserved Domain Architecture
TR
DNA-binding
OCS
Input
DNA-binding
RR
REC
DNA-binding
SF
Region X
Region Y
ODP
DNA-binding
